# P29 An unusual case of reactive arthritis

**DOI:** 10.1093/rap/rkac067.029

**Published:** 2022-09-28

**Authors:** Paul Robinson, David McCormick

**Affiliations:** Ulster hospital, Dundonald, United Kingdom; Ulster hospital, Dundonald, United Kingdom

## Abstract

**Introduction/Background:**

This 28-year-old gentleman with a background of cerebral palsy was admitted to hospital feeling generally unwell with pyrexia as well as a red, hot and swollen right knee. He had communication and mobilty issues at baseline but was very unsettled and less mobile than normal.

CRP was over 100 and elevated temperatures were recorded. Ultrasound confirmed a small right knee effusion which was subsequently aspirated with no growth on culture. Crystal analysis on this sample was also negative.

Intravenous antibiotics were commenced and the patient was managed as a septic joint with no other obvious source of infection.

**Description/Method:**

Antibiotics led to improvement in temperatures and inflammatory markers. A trial off antibiotics led to return of fever, elevated inflammatory markers and knee symptoms. In addition to this a red, hot and swollen left elbow evolved as well as discomfort in neck and left shoulder. Blood cultures remained negative. Repeat knee aspirate remained negative on culture.

Antibiotics were restarted.

An additional problem during admission was constipation which had been a chronic issue that had been worsened.

Abdominal xray showed a 10cm faecolith in the rectum. The case was discussed with the gastro intestinal and surgical teams and conservative management was recommended with escalation of laxatives with only minimal success.

MRI spine showed no discitis. MRI knee showed moderate knee effusion but no osteomyelitis. CRP fluctuated with joint flares over the coming weeks with antibiotics adjusted accordingly.

CT chest abdomen and pelvis to look for an alternative infective source showed massive distention of sigmoid colon with partially calcified faeculant material, with mass effect on the urinary bladder. An infective source was not definitively identified.

The case was discussed with the surgical team again with further usage of enemas to little effect. Ultimately the patient had a manual evacuation performed under anaesthetic in theatre with large volumes of faeces removed. Bowels moved well with ongoing laxatives and his joints remained settled from this point on.

**Discussion/Results:**

This patient had been in hospital for 10 weeks in 2019 with recurring bouts of inflammation in knee and elbow alongside pyrexias and elevated inflammatory markers. Within 48 hours of manual evacuation of a large, chronic faecolith his joints were completely settled and patient was discharged to have no further joint issues since.

The joint issues were initially felt to be an infective aetiology and therefore managed with antibiotics but with the discovery of the large faecolith and subsequent removal of this and settling of joints, this appears to have been a inflammatory arthritis presumably reactive to the chronic constipation.

**Key learning points/Conclusion:**

This was a very interesting case in a young man with physical and mental disabilities with joint pain and swelling and a prolonged hospital admission. Chronic constipation appears to have been the trigger for these inflammatory joints and highlights the point to consider less common causes of inflammatory, reactive arthritis.

